# An Agreement Study Between Point-of-Care and Laboratory Activated Partial Thromboplastin Time for Anticoagulation Monitoring During Extracorporeal Membrane Oxygenation

**DOI:** 10.3389/fmed.2022.931863

**Published:** 2022-06-29

**Authors:** Yuan Teng, Shujie Yan, Gang Liu, Song Lou, Yang Zhang, Bingyang Ji

**Affiliations:** ^1^Department of Cardiopulmonary Bypass, National Center for Cardiovascular Diseases, Fuwai Hospital, Peking Union Medical College, Chinese Academy of Medical Sciences, Beijing, China; ^2^Center of Laboratory Medicine, National Center for Cardiovascular Diseases, Fuwai Hospital, Peking Union Medical College, Chinese Academy of Medical Sciences, Beijing, China

**Keywords:** activated partial thromboplastin time, point of care, extracorporeal membrane oxygenation, anticoagulation, unfractionated heparin

## Abstract

**Background:**

Laboratory activated partial thromboplastin time (LAB-aPTT) is a widely used laboratory assay for monitoring unfractionated heparin (UFH) therapy during extracorporeal membrane oxygenation (ECMO). But LAB-aPTT is confined to a central laboratory, and the procedure is time-consuming. In comparison, point-of-care aPTT (POC-aPTT) is a convenient and quick assay, which might be a promising method for anticoagulation monitoring in ECMO. This study was aimed to evaluate the agreement between POC-aPTT (hemochron Jr. Signature instruments) and LAB-aPTT for anticoagulation monitoring in adult ECMO patients.

**Methods:**

Data of ECMO-supported adult patients anticoagulated with UFH in our institute from January 2017 to December 2020 was retrospectively reviewed. POC-aPTT and LAB-aPTT results measured simultaneously were paired and included in the analysis. The correlation between POC-aPTT and LAB-aPTT was assessed using Spearman’s correlation coefficient. Bias between POC-aPTT and LAB-aPTT were described with the Bland-Altman method. Influence factors for bias were identified using multinomial logistic regression analysis.

**Results:**

A total 286 pairs of aPTT results from 63 patients were included in the analysis. POC-aPTT and LAB-aPTT correlated weakly (*r* = 0.385, *P* < 0.001). The overall bias between POC-aPTT and LAB-aPTT was 7.78 [95%CI (−32.49, 48.05)] s. The overall bias between POC-aPTT and LAB-aPTT ratio (to normal value) was 0.54 [95%CI (−0.68, 1.76)]. A higher plasma fibrinogen level [OR 1.353 (1.057, 1.733), *P* = 0.017] was associated with a higher chance of POC-aPTT underestimating LAB-aPTT. While a lower plasma fibrinogen level [OR 0.809 (0.679, 0.963), *P* = 0.017] and lower UFH rate [OR 0.928 (0.868, 0.992), *P* = 0.029] were associated with a higher chance of POC-aPTT overestimating LAB-aPTT.

**Conclusion:**

The present study showed poor agreement between POC-aPTT and LAB-aPTT. POC-aPTT was not suitable for anticoagulation monitoring in adult ECMO patients.

## Introduction

Extracorporeal membrane oxygenation (ECMO) provides effective respiratory and circulatory support for patients with refractory respiratory failure or cardiogenic shock, improving the survival of these critically ill patients (1–3). In recent years, with the advancement of management and technology, ECMO has been increasingly utilized worldwide. Exposure of blood to non-biological surfaces and the shear stresses of the ECMO circuit activate the coagulation system. Initial fibrinogen deposition and subsequent activation of coagulation factors and complement allow platelets and leucocytes to adhere to the circuit surfaces and enhance thrombin generation (4, 5). Anticoagulation is required to prevent clot formation in this setting. Unfractionated heparin (UFH) is the most commonly used anticoagulant.

Point-of-care (POC) activated clotting time (ACT) is the most convenient and commonly used method for anticoagulation monitoring in ECMO. However, the correlation between ACT and heparin concentration is poor (6, 7). Laboratory activated partial thromboplastin time (LAB-aPTT) is a plasma-based assay and is considered superior to the point-of-care ACT as it shows a better correlation with UFH concentration (8). However, LAB-aPTT is confined to the central laboratory, and the procedure is time-consuming.

Several commercial point-of-care aPTT (POC-aPTT) instruments, including Hemochron Jr. Signature (Accriva Diagnostics, Inc., United States), could offer bedside POC-aPTT results within 3 min, making it a promising method for anticoagulation monitoring during ECMO. Hemochron Jr. Signature aPTT is a whole blood test, and the plasma aPTT is converted and displayed based on the whole blood result (9). The 2021 ELSO anticoagulation guideline mentioned that POC-aPTT test was available but with very limited studies on ECMO (10).

This study was aimed to evaluate the agreement between Hemochron Jr. Signature POC-aPTT and LAB-aPTT test.

## Materials and Methods

### Patients

This was a single-center retrospective study. The study was approved by the institutional ethics board of Fuwai Hospital (NO.2021-1496). The requirement for written informed consent was waived.

This study retrospectively reviewed the clinical data of consecutive adult veno-arterial ECMO (VA-ECMO) patients at Fuwai Hospital from January 2017 to December 2020. Patients who had POC-aPTT and LAB-aPTT measured simultaneously were included in the study. Exclusion criteria were as follows: age < 18 years old, ECMO running time < 48 h, inter-hospital transfer on ECMO, using other anticoagulants (argatroban or bivalirudin).

### Extracorporeal Membrane Oxygenation Management

The indication for VA-ECMO was refractory cardiogenic shock or acute heart failure despite maximum vasoactive agents (VIS > 40) and adequate volume therapy, with at least one of the following indexes: cardiac index < 1.8 L/min/m^2^; left atrial pressure or pulmonary capillary wedge pressure > 20 mmHg; systolic arterial blood pressure < 90 mmHg or mean arterial pressure < 60 mmHg; urine output < 0.5 mL/kg/h, and uncorrectable/continuous metabolic acidosis. The contraindications were (1) severe irreversible neurological injury; (2) irreversible cardiac failure if transplantation or long-term VAD was not considered; (3) contraindication to anticoagulation; (4) uncontrolled surgical massive bleeding. The decision to initiate ECMO was made by a multidisciplinary ECMO team consisting of cardiologists, cardiac surgeons, intensivists, and perfusionists. Femoral-femoral cannulation was preferred in our institute. Central cannulation was chosen when femoral access was difficult or when the patient was complicated with respiratory failure.

MAQUET BE PLS 2050 circuit was used. ECMO flow was initially set at 50∼70 ml/(kg⋅min) and then adjusted to maintain hemodynamic stability and sufficient oxygen supply. UFH was the standard anticoagulant. For non-cardiotomy patients, 50–100 units/kg UFH was given to achieve a goal of ACT 180–200 s before cannulation. For post-cardiotomy patients, UFH was not given until bleeding was controlled with ACT or aPTT below target ranges.

ACT and POC-aPTT were monitored during ECMO every 3 h, while LAB-aPTT was monitored two to four times a day. UFH was titrated according to the above tests and the hemostatic status of the patients. Generally, aPTT goal was 50–80 s. UFH infusion rate was increased or decreased by 1–2 units/kg/h when aPTT was below 50 s or above 80 s, respectively. In situations of bleeding, the goal was 50–60 s. When clots were observed in the oxygenator and the risk of bleeding was low, the goal was 70–80 s or even higher.

Red blood cells (RBC) were transfused when hemoglobin was below 80 g/L. Platelet transfusion trigger was 50 × 10^9/^L. Fibrinogen was given when the fibrinogen level was below 150 mg/dL. Fresh frozen plasma (FFP) was indicated when antithrombin III level was below 50% or INR was below 1.5.

### Point of Care Activated Partial Thromboplastin Time Test

POC-aPTT was performed immediately after the blood sample was drawn from the patient using Hemochron Jr. Signature instrument (Accriva Diagnostics, Inc., United States). 50 μl of whole blood was disposed in the well of a specific 37^°^Cprewarmed size-use cartridge. The equipment automatically drew 15 μl of whole blood into the test tube. After mixing with the reagents, the sample was then exposed to optical detectors. The formation of a clot was indicated to the detectors by a slowing of the flow in the chamber. An internal chronometer linked to the detectors measured the time required to form the clot. POC-aPTT test cuvette was a self-contained disposable test chamber preloaded with a dried preparation of kaolin, phospholipid, stabilizers and buffers. The instrument reported plasma equivalent values mathematically converted from whole blood test results. The normal range of POC-aPTT was 23.2–38.7 s.

### Laboratory Activated Partial Thromboplastin Time Test

LAB-aPTT was measured within 4 h after the blood sample was drawn from the patient. Before the test, the blood sample was stored at room temperature. LAB-aPTT was performed using Stago STA-R Evolution coagulation analyzer and original reagents (STA^®^PTTA, Stago, France). Reagents contained cephalin prepared from rabbit cerebral tissues and a particulate activator (silica) in a buffered medium, lyophilized. The time of fibrin formation was measured in the absence of cellular components by adding activators (silica), calcium and phospholipids to plasma samples. In addition, laboratory staff regularly carried out quality control of LAB-aPTT testing instruments and reagents (STA^®^—System Control N + P). The normal range was 28.5–43.5 s.

### Data Collection and Categories

Data of the patients were retrospectively collected from the electronic medical record system, including demographics, indications, ECMO information, coagulation parameters and outcomes. LAB-aPTT and POC-aPTT tests of samples drawn at the same time were paired and included in the analysis. Laboratory test results of samples drawn at the same time and UFH dose were also collected.

Pairs of LAB-aPTT and POC-aPTT tests were classified into three bias categories depending on bias between POC aPTT and LAB aPTT value: (1) underestimate category: bias <−10 s; (2) accurate category: bias −10∼10 s; (3) overestimate category: bias >10 s.

### Statistical Analysis

Continuous variables with normal distribution were presented as mean ± standard deviation and compared using the one-way ANOVA test. Continuous variables with abnormal distribution were presented as median (interquartile range) and compared using the Kruskal-Wallis test.

The agreement between POC-aPTT and LAB-aPTT for anticoagulation monitoring was assessed step by steply.

Firstly, correlations among LAB-aPTT, POC-aPTT and UFH doses were evaluated using the Spearman correlation coefficient.

Secondly, the Bland and Altman plots method was performed to describe biases between LAB-aPTT and POC-aPTT. The limits of agreement between LAB-aPTT and POC-aPTT were presented as bias (1.96 SD). aPTT values and aPTT ratios of the values to normal control were analyzed separately.

The Kruskal-Wallis test and multinomial logistic regression analysis were employed to analyze the association between biases (classified into three bias categories as mentioned above) and a set of variables including blood hemoglobin level, blood platelet count, plasma fibrinogen level, plasma D-dimer level, plasma antithrombin activity (AT), plasma prothrombin time (PT), and UFH dose. The variables were chosen because they were related to the coagulation system, and they were measured from the same blood sample with LAB aPTT. Only statistically significant variables in Kruskal-Wallis tests were used as covariates in multinomial logistic regression analysis. The reference category for the outcome variable was “accurate,” and each of the other two categories was compared to this reference group.

Thirdly, the predictive performance of POC-aPTT for guiding UFH dose titration was evaluated, taking LAB-aPTT as the gold-standard method. The goals of aPTT varied across centers. aPTT could be maintained within the range of 50–80 s or 1.5–2.5 times normal, according to expert recommendations (10–12). Positive predictive value (PPV) and negative predictive value (NPV) for diagnosing an aPTT <50 s (or < 1.5 times normal), 50–80 s (or 1.5–2.5 times normal) and >80 s (or >2.5 times normal) were calculated, respectively.

Statistical analysis was performed using SPSS 25.0 (IBM Corp., Chicago, IL, United States). For all analyses, 2-tailed *p* < 0.05 was considered statistically significant.

## Results

### Patient Characteristics

**FIGURE 1:**
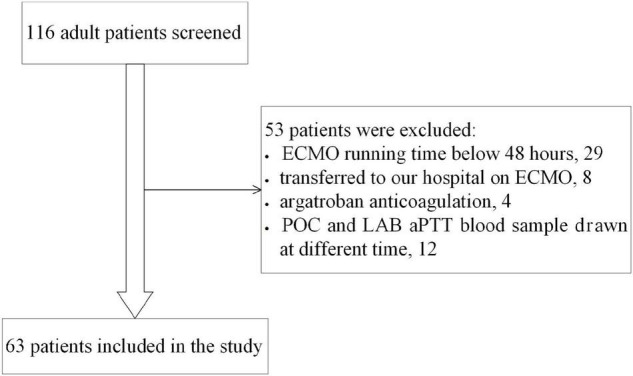
Flowchart of patient inclusion.

Sixty-three eligible patients were included in the study (Figure 1), with 286 pairs of aPTT tests included in the agreement analysis. Forty-six patients (73%) were in the post-cardiotomy group. 30-day survival rate before discharge was 60.3%. Data of the patients are shown in Table 1.

**TABLE 1:** Patient characteristics (*n* = 63).

Variables	All patients (*n* = 63)
**Demographic data**	
Age (year)	47.49 ± 13.12
Male, *n* (%)	36 (57.1%)
Height (cm)	166.6 ± 8.79
Weight (kg)	65.45 ± 12.75
**Indication**	
Postcardiotomy, *n* (%)	46 (73%)
Non-postcardiotomy, *n* (%)	17 (27%)
**Laboratory values at 6 h after ECMO initiation**	
Hemoglobin (g/L)	94 (81.5, 109.3)
Platelets (10^9^/L)	88.5 (56.3, 149.3)
Creatinemia (μmol/L)	135.5 (116.1, 163.1)
Prothrombin time (s)	18.75 (16.28, 23.63)
Total bilirubin (μmol/L)	37.09 (22.88, 54.35)
Lactate (mmol/L)	6.75 (3.78, 11.10)
**Outcome**	
30-day Mortality, *n* (%)	25 (39.7%)
Duration of ECMO (hours)	153 (96, 192)
Mechanical ventilation time (days)	9 (6, 16)
Duration in the ICU (days)	18 (10, 27)
Length of stay (days)	33 (21, 45)
CRRT, *n* (%)	27 (42.9%)
Red blood cells transfusion during ECMO (u)	10 (4, 14)
Fresh frozen plasma transfusion during ECMO (ml)	800 (400, 1,800)
Platelet transfusion during ECMO (u)	1 (0, 3)

*ECMO, extracorporeal membrane oxygenation; CRRT, continuous renal replacement therapy; ICU, intensive care unit.*

### Correlations Among Laboratory Activated Partial Thromboplastin Time, Point of Care Activated Partial Thromboplastin Time, and Unfractionated Heparin Doses

**FIGURE 2:**
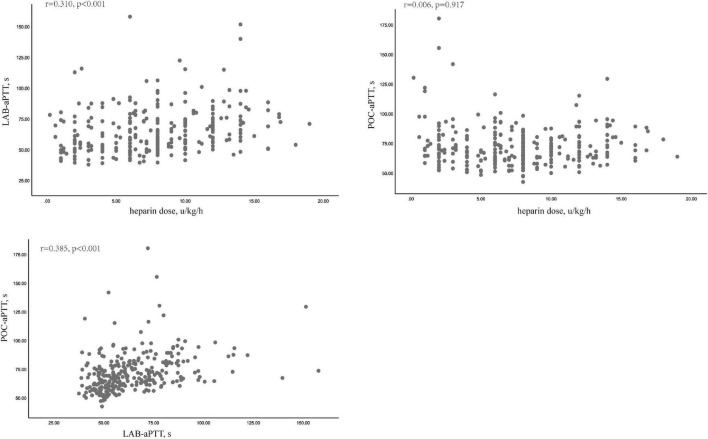
Correlations among LAB-aPTT, POC-aPTT and UFH doses.

POC-aPTT and LAB-aPTT correlated weakly (*r* = 0.385, *P* < 0.001). UFH dose and LAB-aPTT correlated weakly (*r* = 0.31, *P* < 0.001). There was no correlation between UFH dose and POC-aPTT (*r* = 0.006, *P* = 0.917). Scatterplots of these data were depicted in Figure 2.

### Bias Between Point of Care Activated Partial Thromboplastin Time and Laboratory Activated Partial Thromboplastin Time

**FIGURE 3:**
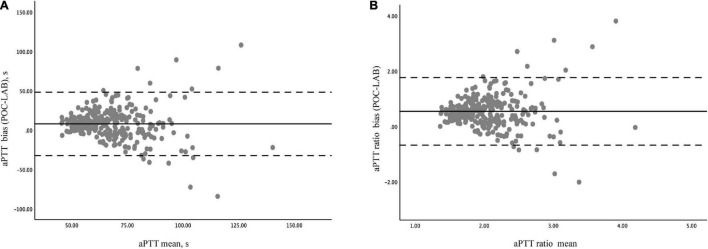
Bland –Altman diagram of the difference and agreement between POC-aPTT and LAB-aPTT. **(A)** The total bias between POC-aPTT and LAB-aPTT value. POC-LAB: POC-aPTT value—LAB-aPTT value, aPTT mean: 1/2 × (POC-aPTT value + LAB-aPTT value). **(B)** The total bias between POC-aPTT and LAB-aPTT ratio. POC-LAB ratio: POC-aPTT ratio to the normal value—LAB-aPTT ratio to the normal value, aPTT ratio mean: 1/2 × (POC-aPTT ratio to the normal value + LAB-aPTT ratio to the normal value). The bias representatives the systematic error between the two judgments (bold line), the mean difference ± 1.96 standard deviations represents the limit of agreement or the 95% confidence interval (dotted line).

Bland- Altman analysis for 286 pairs of aPTT tests showed that the overall bias between POC-aPTT and LAB-aPTT was 7.78 [95%CI (−32.49, 48.05)] s (Figure 3A). The overall bias between POC-aPTT and LAB-aPTT ratio (to normal value) was 0.54 [95%CI (−0.68, 1.76)] (Figure 3B).

Bland-Altman analyses were performed in postcardiotomy and non-postcardiotomy groups, respectively, to evaluate the influence of cardiac surgery on the consistency of the two methods. The biases of aPTT value in the postcardiotomy group and the non-postcardiotomy group were 5.06 [95%CI−28.82, 38.94)] s and 13.08 [95%CI (−34.84, 60.99)] s, respectively. The biases of aPTT ratio in postcardiotomy group and non-postcardiotomy group were 0.45 [95%CI (−0.55, 1.45)] and 0.70 [95%CI (−0.75, 2.15)], respectively (Supplementary Figure 1).

### Influence Factors for Biases

Blood hemoglobin level, blood platelet count, plasma fibrinogen level, plasma D-dimer level, AT activity, PT, UFH dose, and LAB-aPTT were compared among the three bias categories (Table 2). Three statistically significant covariates (plasma fibrinogen level, AT activity, UFH dose) were put into the multinomial logistic regression analysis. A higher plasma fibrinogen level [OR 1.353 (1.057, 1.733), *P* = 0.017] was associated with a higher chance of POC-aPTT underestimating LAB-aPTT. While a lower plasma fibrinogen level [OR 0.809 (0.679, 0.963), *P* = 0.017] and a smaller UFH dose [OR 0.928 (0.868, 0.992), *P* = 0.029] were associated with a higher chance of POC-aPTT overestimating LAB-aPTT (Table 3).

**TABLE 2:** Univariate analysis for bias categories.

Variables	Total	Underestimate bias <-10 (*n* = 36)	Accurate -10 ≤ bias ≤ 10 (*n* = 120)	Overestimate bias>10 (*n* = 130)	*P*-value
Hemoglobin (g/L)	92 (87,102)	93.5 (88,100)	91 (86,96.5)	91.5 (86,107)	0.187
Platelets (10^9^/L)	64.5 (47,91)	72 (50.5,102)	60 (47.5,81.5)	58.5 (43,87)	0.270
Fibrinogen (g/L)	4.09 (2.94,5.39)	5.335 (4.40,6.30)	4.43 (3.55,5.70)	3.875 (2.82,4.54)	**0.000**
D-dimer	4.32 (2.23,7.59)	4.585 (3.09,7.37)	3.5 (1.97,6.64)	5.03 (2.43,9.27)	0.130
AT (%)	64 (46.25,78)	73 (61,87)	65 (54.5,79)	61.5 (43,80)	**0.001**
PT (s)	15.8 (14.6,18.6)	15.9 (14.9,17.6)	15.6 (14.4,17.7)	15.75 (14.5,18.8)	0.176
INR	1.26 (1.14,1.57)	1.265 (1.16,1.45)	1.24 (1.12,1.48)	1.26 (1.14,1.59)	0.126
UFH dose (u/kg/h)	8 (4.6,10)	8 (7,11.3)	8 (5.65,10.8)	6.55 (2,10)	**0.000**

*AT, antithrombin; PT, prothrombin time; INR, international normalized ratio; UFH, unfractionated heparin; LAB-aPTT, laboratory activated partial thromboplastin time. Bold values indicate P values with statistically significant.*

**TABLE 3:** Multinomial logistic regression analysis for bias categories.

Covariates	Underestimate vs. accurate					Overestimate vs. accurate				
	Estimate (SE)	OR	95% Wald CL for OR		*P*-value	Estimate (SE)	OR	95% Wald CL for OR		*P*-value
			Lower	Upper				Lower	Upper	
Fibrinogen (g/L)	0.302 (0.126)	1.353	1.057	1.733	**0.017**	−0.212 (0.089)	0.809	0.679	0.963	**0.017**
AT (%)	0.013 (0.011)	1.014	0.992	1.035	0.218	−0.008 (0.007)	0.992	0.979	1.005	0.241
UFH dose (u/kg/h)	0.027 (1.033)	1.027	0.924	1.142	0.615	−0.075 (0.034)	0.928	0.868	0.992	**0.029**

*SE, standard error; OR, odds ratio; AT, antithrombin; LAB-aPTT, laboratory activated partial thromboplastin time; POC-aPTT, point of care activated partial thromboplastin time. Bold values indicate P values with statistically significant.*

### Predictive Performance of Point of Care Activated Partial Thromboplastin Time on Unfractionated Heparin Doses Titration

Supplementary Tables 1, 2 showed distributions of aPTT results according to target ranges. Taking LAB-aPTT as a gold method, the predictive performance of POC-aPTT to accurately guide UFH dose titration was very poor. The diagnostic PPV and NPV of POC-aPTT to diagnose an aPTT <50 s (or < 1.5 times normal), 50–80 s (or 1.5–2.5 times normal) and > 80 s (or > 2.5 times normal) were listed in Table 4.

**TABLE 4:** Positive predictive value (PPV) and negative predictive value (NPV) of POC-aPTT taking LAB-aPTT as the gold standard method.

	PPV	NPV
POC-aPTT value		
<50 s	80.0%	77.9%
50–80 s	63.0%	50.0%
>80 s	36.9%	88.7%
POC-aPTT ratio to normal control		
<1.5	100%	66.3%
1.5–2.5	54.2%	28.2%
>2.5	11.9%	95.5%

## Discussion

The present study was aimed to explore the agreement between POC-aPTT and LAB-aPTT for anticoagulation monitoring during ECMO. The results showed discordance between the two assays. Firstly, POC-aPTT and LAB-aPTT correlated weakly (*r* = 0.385, *P* < 0.001). No correlation was found between POC-aPTT and UFH dose. Secondly, the bias between POC-aPTT and LAB-aPTT was large (7.78 [95%CI (−32.49, 48.05)]). Thirdly, the agreement for guiding UFH titration between the two methods was poor.

The result of our study was similar to Ferring’s study, which reported a large bias (17 ± 33.1 s) between POC-aPTT (CoaguCheck^®^ Pro, Boehringer Mannheim Diagnostics, United States, Roche Diagnostics, Switzerland) and LAB-aPTT in surgical intensive care patients following cardiovascular or major abdominal surgery (13). Gauss et al. reported the bias between POC-aPTT (Hemochron Jr. Signature instruments) and LAB-aPTT ratio was 1.13 in patients with acute hemorrhage. 89% of the POC-aPTT values exceeded the predefined limits of agreement (9).

Methodological differences account for poor agreement between POC-aPTT and LAB-aPTT, with different reagents and equipment. POC-aPTT test reagents are phospholipids and kaolin, while LAB-aPTT uses silicon dioxide, ellagic acid, calcium and phospholipid. The differences in reagents also account for the discordance between point-of-care viscoelastic coagulation tests in ECMO. Giani et al. (14) compared rotational thromboelastometry (ROTEM) INTEM assay, kaolin thromboelastography (TEG) and LAB-aPTT for ECMO anticoagulation monitoring and found that correlation with LAB-aPTT was higher for INTEM clotting time (*R*^2^ = 0.34, *P* < 0.001) compared with Kaolin TEG R time (*R*^2^ = 0.08, *P* = 0.014). A potential explanation was that compared to kaolin TEG, the reagent of INTEM was more similar to LAB-aPTT. The activator in Kaolin TEG assay is kaolin, while INTEM (ROTEM) activators are phospholipid and ellagic acid. The pre-analysis variables such as collection tube citrate concentration might incur variations between the two techniques (15).

Secondly, the results of the POC-aPTT test are not actually derived from plasma, which instead are obtained *via* whole blood tests and converted to plasma aPTT levels with an algorithm. The results of POC-aPTT are theoretically calibrated based on normal hematocrit and platelet count (16). Hemodilution, high shear force, hemolysis, UFH anticoagulation, and surgical bleeding during ECMO can decrease hemoglobin and platelet counts (17, 18). A study showed that the changes in hematocrit and platelet levels of blood samples might affect POC aPTT results (16), although the current study did not find the association of hematocrit and platelet with aPTT bias. Furthermore, the manufacturer’s specification also recommends that blood samples with HCT < 20% should not be used for POC-aPTT measurement because the optical density of the sample is beyond the detection range of the instrument. In our study group, only 10% of the blood sample had blood hemoglobin levels below 8 g/dl.

Thirdly, the activation of blood components and coagulation pathway factors during ECMO might also affect the agreement of the two methods. In our study, we found that fibrinogen affected the bias between POC-aPTT and LAB-aPTT. A recent study also found that increased fibrinogen, FVIII, FXI, and FXII levels in ICU patients attenuated the association between POC-aPTT and LAB-aPTT (19). Toulon found the agreement between POC-aPTT (CoaguChek™ Pro DM) and LAB-aPTT was unacceptable in patients undergoing bleeding surgery (20), and the bias of POC and LAB -aPTT increased with the increasing severity of coagulopathy. Considering cardiac surgery may lead to coagulopathy (21, 22), we performed subgroup Bland-Altman analyses in cardiotomy and non-cardiotomy groups and poor agreement was found in both populations.

In addition, our study found that a smaller UFH dose was associated with a higher chance of POC-aPTT overestimating LAB-aPTT. Therefore, when the UFH infusion rate was low, using POC-aPTT for anticoagulation monitoring would increase the risk of thrombosis.

Although the agreement with LAB-aPTT is poor, POC-aPTT is still of some value during ECMO, especially when timely anticoagulation monitoring is required or during ECMO transport when LAB-aPTT is not available. The turnaround time of POC-aPTT is much shorter than LAB-aPTT. Lardinois reported that the turnaround time of LAB-aPTT was 92.0 min (IQR, 69.3–121.2), much longer than that of POC-aPTT (*P* < 0.0001) (19). A shorter turnaround time could help reduce thrombotic and hemorrhagic complications by avoiding insufficient or excessive anticoagulation, especially in the early stage of the ECMO run. PPV for diagnosing an aPTT value below 50 s was 80% in our study. Another study found a good correlation between LAB-aPTT and POC-aPTT when the results were < 60 s (23). Therefore, POC-aPTT could help identify insufficient anticoagulation quickly and prevent thrombosis in the ECMO circuit, which is essential in ECMO transport and during massive blood transfusions.

This study had some limitations. First of all, it was a retrospective study. Some essential variables were not available for analysis, including anti-Xa and coagulation factors. In our institute, coagulation factors were not routinely measured. Anti-Xa was not used for anticoagulation monitoring in ECMO patients until recently. Second, measurement failure could not be excluded. Third, only VA-ECMO patients were included in the study as our institute was a specialized cardiac center. Moreover, in the predictive performance analysis, aPTT goal was not determined according to the anti-Xa test locally. Finally, we did not compare POC-aPTT and LAB-aPTT on bleeding and thrombosis events as it was tough to retrospectively determine clinical hemostasis and bleeding status within 1–3 h before aPTT were tested.

## Conclusion

The present study showed poor agreement between POC-aPTT and LAB-aPTT. POC-aPTT was not suitable for anticoagulation monitoring in adult ECMO patients.

## Data Availability Statement

The original contributions presented in this study are included in the article/Supplementary Material, further inquiries can be directed to the corresponding author/s.

## Ethics Statement

This study was approved by the Institutional Ethics Board of Fuwai Hospital (No.2021-1496), and the individual consent for this retrospective analysis was waived. Written informed consent for participation was not required for this study in accordance with the national legislation and the institutional requirements.

## Author Contributions

YT and SY designed the study, extracted the data, and drafted the manuscript. SY and GL performed data analysis. YZ wrote the sections of the manuscript. SL and BJ revised the manuscript for the final version. All authors contributed to the article and approved the submitted version.

## Conflict of Interest

The authors declare that the research was conducted in the absence of any commercial or financial relationships that could be construed as a potential conflict of interest.

## Publisher’s Note

All claims expressed in this article are solely those of the authors and do not necessarily represent those of their affiliated organizations, or those of the publisher, the editors and the reviewers. Any product that may be evaluated in this article, or claim that may be made by its manufacturer, is not guaranteed or endorsed by the publisher.

## Abbreviations

VA-ECMO: veno-arterial extracorporeal membrane oxygenation
LAB-aPTT: laboratory activated partial thromboplastin time
POC-aPTT: point of care activated partial thromboplastin time
UFH: unfractionated heparin
ACT: activated clotting time
VIS: maximum vasoactive agents
ICU: intensive care unit
PPV: positive predictive value
NPV: negative predictive value.
