# Supplementary material for: An Agreement Study Between Point-of-Care and Laboratory Activated Partial Thromboplastin Time for Anticoagulation Monitoring During Extracorporeal Membrane Oxygenation
Source: Front Med (Lausanne). 2022 Jun 29;9:931863. doi: 10.3389/fmed.2022.931863 (PMC9276956; doi:10.3389/fmed.2022.931863)
Supplement: Supplementary file 1 [file Data_Sheet_1.zip › Supplementary Table 2.docx]

Supplementary Table 2. Distributions of aPTT ratio according to target anticoagulation monitor range.

|  |  | LAB aPTT ratio (to normal control) | | |
| --- | --- | --- | --- | --- |
|  |  | ＜1.5 | 1.5-2.5 | ＞2.5 |
| POC aPTT ratio  (to normal control ) | ＜1.5 | 1 | 0 | 0 |
|  | 1.5-2.5 | 83 | 109 | 9 |
|  | ＞2.5 | 13 | 61 | 10 |
