# Supplementary material for: An Agreement Study Between Point-of-Care and Laboratory Activated Partial Thromboplastin Time for Anticoagulation Monitoring During Extracorporeal Membrane Oxygenation
Source: Front Med (Lausanne). 2022 Jun 29;9:931863. doi: 10.3389/fmed.2022.931863 (PMC9276956; doi:10.3389/fmed.2022.931863)
Supplement: Supplementary file 1 [file Data_Sheet_1.zip › Supplementary Figure legend.docx]

**Supplementary Figure 1.** Bland–Altman diagram between the POC-aPTT and LAB-aPTT in postcardiotomy group and non-postcardiotomy group.
