# Supplementary material for: An Agreement Study Between Point-of-Care and Laboratory Activated Partial Thromboplastin Time for Anticoagulation Monitoring During Extracorporeal Membrane Oxygenation
Source: Front Med (Lausanne). 2022 Jun 29;9:931863. doi: 10.3389/fmed.2022.931863 (PMC9276956; doi:10.3389/fmed.2022.931863)
Supplement: Supplementary file 1 [file Data_Sheet_1.zip › Supplementary Table 1.docx]

Supplementary Table 1. Distributions of aPTT value according to target anticoagulation monitor range.

|  |  | LAB aPTT value | | |
| --- | --- | --- | --- | --- |
|  |  | ＜50s | 50-80s | ＞80s |
| POC aPTT value | ＜50s | 4 | 1 | 0 |
|  | 50-80s | 55 | 136 | 25 |
|  | ＞80s | 7 | 34 | 24 |
